# Supplementary material for: Microbiota Metabolism Failure as a Risk Factor for Postoperative Complications after Aortic Prosthetics
Source: Biomedicines. 2023 Apr 30;11(5):1335. doi: 10.3390/biomedicines11051335 (PMC10216268; doi:10.3390/biomedicines11051335)
Supplement: Supplementary file 1 [file biomedicines-11-01335-s001.zip › Table S1.pdf]

Table S1. Frequency of concomitant diseases in patients with aortic aneurysm/dissection included in the study (n=79)

**Concomitant heart diseases**	Patients, n	%
Heart defects	55	70
Multifocal atherosclerosis	24	30
Coronary heart disease	20	25
Hypertension	57	72
Cardiac arrhythmias: atrial fibrillation, atrial flutter, or ventricular extrasystole	14	18
Conduction disturbances	4	5
**Other concomitant diseases**		
Connective tissue dysplasia	10	12
Chronic kidney disease	16	20
Type II diabetes mellitus	7	9
Diseases of the gastrointestinal tract, including gastritis, duodenitis, gastroduodenitis, gastric ulcer and/or duodenal ulcer in remission	48	61
Obesity of any degree	12	15
