# Supplementary figures and images for: Microbiota Metabolism Failure as a Risk Factor for Postoperative Complications after Aortic Prosthetics
Source: Biomedicines. 2023 Apr 30;11(5):1335. doi: 10.3390/biomedicines11051335 (PMC10216268; doi:10.3390/biomedicines11051335)

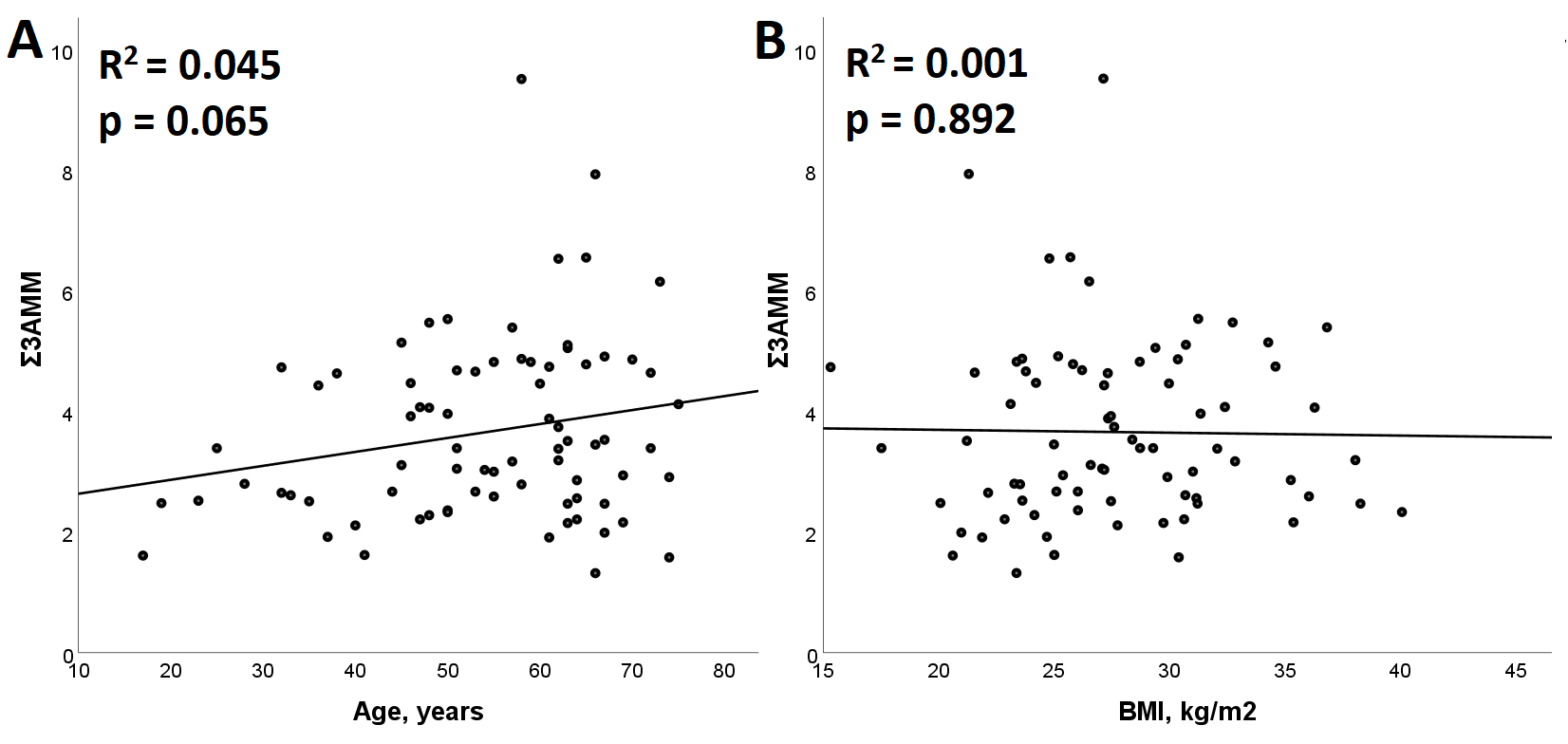

Supplement: Supplementary file 1 [file biomedicines-11-01335-s001.zip › Figure S2.png]
